# Demographic, dietary and physical activity predictors of general and abdominal obesity among university students: a cross-sectional study

**DOI:** 10.1186/s40064-015-0999-2

**Published:** 2015-05-15

**Authors:** Victor Mogre, Rauf Nyaba, Samuel Aleyira, Napoleon B. Sam

**Affiliations:** Department of Human Biology, School of Medicine and Health Sciences, University for Development Studies, P.O. Box TL 1883, Tamale, Ghana; Department of Allied Health Sciences, School of Medicine and Health Sciences, University for Development Studies, P.O. Box TL 1883, Tamale, Ghana; Academic Affairs Unit, University for Development Studies, P.O. Box 1350, Tamale, Ghana

**Keywords:** Abdominal obesity, Physical activity, Dietary habits, University students, General obesity, Tamale, Ghana

## Abstract

**Background:**

Obesity has become a disease of global public health concern in both developing and developed countries. We investigated the influence of socio-demographic, dietary habits and physical activity levels on general and abdominal obesity among a sample of university students in Ghana.

**Methods:**

This cross-sectional study was carried out among a sample of 552 students attending the University for Development Studies, School of Medicine and Health Sciences, Tamale, Ghana. Demographic characteristics were assessed using questionnaire. Dietary habits were measured by means of food frequency questionnaires. Anthropometric measurements were done using appropriate methods. Physical activity levels were measured using the World Health Organization (WHO) global physical activity questionnaire. Spearman’s nonparametric correlation coefficient and multinomial logistic regression analysis were used to investigate the determinants of general and abdominal obesity.

**Results:**

The prevalence of general overweight/obesity (25.8 % *vs.* 5.9 %) and abdominal obesity (40.9 % *vs.* 0.8 %) was higher in female students than in male students. General overweight/obesity was less likely in students who engaged in vigorous physical activity (Adjusted Odds Ratio (AOR) = 0.3, 95 % CI = 0.1 – 0.7, p = 0.004), but more likely in students who consumed fruits and vegetables > 3 days per week (AOR = 2.6, 95 % CI = 1.2 – 5.4, p = 0.015). Abdominal obesity was also less likely in male students (AOR = 0.0, 95 % CI = 0.0 – 0.5, p = 0.017) but more likely in students who consumed roots and tubers > 3 times per week (AOR = 8.0, 95 % CI = 2.2 – 10.1, p = 0.017) and in those who consumed alcoholic and non-alcoholic beverages > 3 times per week (AOR = 8.2, 95 % CI = 2.2 – 31.1, p = 0.002).

**Conclusion:**

Demographic factors, dietary habits and physical activity levels were found to be associated to general overweight/obesity and abdominal obesity. General overweight/obesity was associated to the consumption of fruits and vegetables > 3 times a week. The consumption of roots and tubers > 3 times per week and alcoholic and non-alcohol beverages > 3 times a week was associated to abdominal obesity. Engagement in vigorous physical activity and being male were negatively associated to general overweight/obesity and abdominal obesity respectively. Promotion of active lifestyles in young adults should be encouraged.

## Background

Globally, obesity has become an epidemic occurring in both developed and developing countries (World Health Organization 1997; James et al. 2001). Recent findings reported from Ghana and other countries in sub-Saharan Africa have shown that obesity is becoming increasingly common in both young (Mogre et al. 2014; Amidu et al. 2013; Oghagbon et al. 2009; Olusanya and Omotayo 2011; Onyechi and Okolo 2008) and older adults (Kamadjeu et al. 2006; Mogre et al. 2012).

It’s been projected that by the year 2020, the global impact of non-communicable diseases will cause up to 73 % of deaths and 60 % of the disease burden (Mufunda et al. 2006). Obesity and its co-morbidities are the leading causes of such non-communicable diseases including cardiovascular disease (CVD), type 2 diabetes, some types of cancer and the metabolic syndrome (Visscher and Seidell 2001).

Even though BMI-measured obesity has been shown to be associated to cardiovascular diseases and other forms of non-communicable diseases, some studies report that pattern of body (Wei et al. 1997; Folsom et al. 1993; Xavier Pi-Sunyer 2000) fat distribution is a more important determinant than general obesity. Abdominal obesity measured by waist circumference has been shown to be associated with increased overall risk of cardiovascular diseases and diabetes (Prineas et al. 1993).

Despite the strong relationship between obesity and genetics, several other factors have been identified for the rising prevalence of overweight and obesity in developing countries including nutrition transition and sedentary lifestyle (Mogre et al. 2014; Al-Hazzaa et al. 2012; Azadbakht and Esmaillzadeh 2008). Unhealthy dietary habits have been associated with obesity and several chronic diseases such as cardiovascular diseases and cancer (Parkin 2011; Reddy and Katan 2004). The risk of developing chronic diseases has been shown to increase with the consumption of a diet rich in energy, total fat, saturated fat and cholesterol but relatively low in unsaturated fats, fruits and vegetables. Large epidemiological studies have also demonstrated associations between higher physical activity levels and lower rates of several chronic diseases (Warburton et al. 2010; Lee et al. 2012). Socio-demographic factors such as gender, marriage, and educational level, among others, have also been shown to be associated to obesity (Mogre et al. 2014; Mogre et al. 2012). Sedentary lifestyles together with the nutrition transition characterized by the proliferation of fast food spots might affect the dietary habits of young adults in developing countries including Ghana increasing their risk of developing obesity (Mogre et al. 2014; Yahia et al. 2008). However, studies on the dietary habits, physical activity levels and socio-demographic characteristics and their associations to obesity are limited in sub-Saharan Africa including Ghana. Data are needed to provide an increased understanding of these associations for health policy makers and health providers to design programs for the effective prevention and management of obesity in young adults in Ghana.

This study investigated the associations between general overweight/obesity and abdominal obesity and demographic factors (age and sex), dietary habits and physical activity levels in a sample of university students in Ghana.

## Methods

This cross-sectional study was carried out among a sample of 552 students (aged 18–36 years) attending the University for Development Studies, School of Medicine and Health Sciences (UDS-SMHS), Tamale, Ghana. All students of the UDS-SMHS were eligible to participate in the study. From the eligible student population of 1809, the participants of the study were selected using a random number statistical table that yielded a proportionate random sample that included more participants following academic programs with larger student population. Five hundred and sixty-five students agreed to participate in the study. Thirteen questionnaires were incomplete remaining 552 questionnaires yielding a response rate of 98.1 %. Participation in the study was voluntary and informed consent was sought from each participant. Potential participants who self-reported pregnancy or breast-feeding were excluded from the study because of its potential impact on abdominal fat accumulation. The study was approved by the Ethics Committee of the University for Development Studies, School of Medicine and Health Sciences, Ghana.

### Anthropometric measures

Anthropometric measurements of body weight and height were measured without shoes and with light clothing by trained personnel. Weight was measured to the nearest 0.1 kg using a UNICEF electronic scale manufactured by seca. Height was measured using a wall-mounted microtoise and recorded to the nearest 0.5 cm. BMI was calculated as weight (kg)/height^2^ (m^2^) and used to categorize *BMI-measured* weight status: underweight (BMI ≤ 18.5), normal weight (BMI 18.5–24.9), overweight (BMI 25.0–29.9) and obese (BMI ≥ 30) (WHO 2000).

Waist circumference (WC) was measured midway between the inferior angle of the ribs and the suprailiac crest (WHO 2008) to the nearest 0.5 cm using a non-stretchable fibre-glass measuring tape (Butterfly, China). During the measurement, participants stood in an upright position, with arms relaxed at the side, feet evenly spread apart and body weight evenly distributed in accordance with the WHO expert consultation report on waist circumference and waist-to-hip ratio (WHR) (WHO 2008). Abdominal obesity was determined as a waist circumference >102 cm in men and >88 cm in women according to the World Health Organization cut-off points and risk of metabolic complications for Waist circumference (WHO 2008).

### Dietary habits

A food frequency questionnaire (FFQ) was designed to assess the dietary habits of the participants. The FFQ consisted of a list of 38 food items. Participants were asked to report their frequency of consumption (number of times consumed weekly) of the foods listed. The listed foods were put into 7 food groups: cereals and grains (e.g. rice, tuo zaafi (T.Z.) etc.), roots and tubers (yam, plantain, potatoes, etc.), beans and nuts (soya beans, cowpea, groundnuts), fruits and vegetables (mangoes, orange, pawpaw, banana, cabbage, lettuce, carrots, etc.), alcoholic and non-alcoholic beverages (fruit juice, soda drinks, tea, yogurt, beer, wine, whisky, etc.) and fats and oils (palm oil, shea butter, margarine, butter, etc.). The students reported their frequency of consumption per week of the food items in a food group on the basis of 8 responses: rarely or never; once; twice; thrice; four; five; six; and 7 times.

Due to the lack of uniformly accepted portion sizes in Ghana, we excluded quantities of food consumed. Frequency of consumption scores for each of the food items in a food group was calculated. These were used to compute an average frequency of consumption per week for each food group. On the basis of the median and mean scores of the frequency of intake for each of the food groups, a dietary cut off point of > 3 times per week was used to categorize the frequency of intake. A frequency of intake >3 times per week was considered as high intake and < 3 times per week considered low intake. Socio-demographic data were also collected through the FFQ. The FFQ had a Cronbach alpha of 0.71 indicating a good level of reliability.

### Physical activity level

The Global Physical Activity Questionnaire (GPAQ) was used to measure the level of physical activity of the participants (WHO 2009). The GPAQ consist of 16 questions about physical activity level in a typical week. The frequency and duration of time spent doing physical activity is measured in 3 domains: activity at work, travel to and from places and recreational activities.

The GPAQ was used due to its standardization, easiness to administer, relative unobtrusiveness and inexpensiveness. Its reliability and validity has been found to be 0.67–0.81 and 0.54 respectively (Armstrong and Bull 2006). Without modifications, the questionnaire was fully adapted for the study. However, to suit the Ghanaian context, local examples of types and intensity of activities were used. The GPAQ analysis protocol for the collection of all data and processing, was followed (WHO 2009). All activity durations were converted into minutes. Energy expenditure, measured in metabolic equivalents (MET), was estimated using duration, intensity and frequency of physical activities performed within 7 days. MET is the ratio of specific physical activity metabolic rates to the resting metabolic rate (1 MET = the energy cost of sitting quietly, and was equivalent to a caloric consumption of 1 kcal/kg/hour). A MET-minute showed the total activity volume on weekly basis, and was calculated by multiplying time spent on each activity during a week by the MET-values of each level of activity. Using the compendium of physical activities (Ainsworth et al. 2000), MET-values for various levels of activities was established. MET values of 4 and 8 were set for moderate-intensity (transport-related walking or cycling) and vigorous-intensity physical activity, respectively. Total MET/minutes/week was computed by the sum of all moderate- to vigorous-intensity physical activities performed at work, transport and recreation. Based on the total MET/minutes/week, subjects were classified into light, moderate, and vigorous physical activity intensity (PA) as defined by the GPAQ analysis framework (WHO 2009).

#### Vigorous

A participant found within any of the following categories: Vigorous-intensity activity on at least 3 days achieving at least 1,500 MET-minutes/weeks OR 7 or more days of any combination of walking, moderate or vigorous intensity activities achieving at least 3,000 MET-minutes per week.

#### Moderate

A participant not achieving the criteria for the high category but either of the following 3 criteria: (a) 3 or more days of vigorous-intensity of at least 60 mins per day OR (b) 5 or more days of moderate-intensity and/or walking of at least 30 mins per day OR (c) 5 or more days of any combination of walking, moderate-or vigorous- intensity activities accumulating at least 600 MET-minutes/week.

#### Light

Participant’s reported activity is lower than the categories outlined above or no activity is reported at all.

### Statistical analysis

All data were checked and entered into SPSS version 18, which was used for all the data analysis. Descriptive statistics were performed and presented as mean ± sd. Student t-tests were performed to assess the significance of differences between groups in the distribution of continuous variables and Fisher’s exact test for categorical variables. Cronbach alpha was used to test reliability of the food frequency questionnaire. Spearman’s nonparametric correlation coefficients were calculated among all study variables (dietary habits, demographic, physical activity levels and anthropometric variables) to evaluate their associations. Multivariable logistic analyses were conducted to identify predictors of general overweight/obesity and abdominal obesity. Gender and age were adjusted in the initial logistic regression models. Adjusted odds ratios (AOR) at 95 % CIs were calculated for each independent predictor. Independent predictors of adiposity (general overweight/obesity and abdominal obesity) included demographic (male or female, age); dietary habits (consumption > 3 times per week per food group); and physical activity (light, moderate and vigorous). General overweight/obesity was dichotomized as being generally obese or not generally overweight/obese based on the WHO classification previously described. Abdominal obesity was also dichotomized as being abdominal obese or non-abdominally obese based on the cut off points described above. A p-value of less than 0.05 was considered significant.

## Results

Presented in Table 1 are the basic characteristics of the participants.

**Table 1:** Characteristics of the study sample

Variable	Total (n = 552)	Male (n = 354)	Female (n = 198)	P value
Age (years)	23.0 ± 2.8	23.6 ± 2.9	22.1 ± 2.3	<0.001
Age (≥30 years)	21 (3.8 %)	12 (3.4 %)	9 (4.5 %)	
Weight (Kg)	62.3 ± 9.65	63.4 ± 7.5	60.3 ± 12.4	0.002
Height (m)	1.7 ± 0.1	1.7 ± 0.1	1.6 ± 0.1	<0.001
BMI (Kg/m^2^)	22.0 ± 3.2	21.5 ± 2.4	22.8 ± 4.2	<0.001
General overweight/obesity (%)	72 (13.0 %)	21 (5.9 %)	51 (25.8 %)	<0.001
WC (cm)	77.7 ± 6.8	76.6 ± 5.0	79.8 ± 8.7	<0.001
Abdominal obesity (%)	84 (15.2 %)	3 (0.8 %)	81 (40.9 %)	<0.001
Food group				
Cereals and grains (Mean ± SD)	3.1 ± 1.1	3.2 ± 1.2	3.0 ± 1.1	0.011
>3 days/week	204 (37.0 %)	141 (39.8 %)	63 (31.8 %)	0.066
Roots and tubers (mean	1.5 ± 1.2	1.5 ± 1.3	1.5 ± 1.0	0.977
>3 days/week (Mean ± SD)	30 (5.4 %)	24 (6.8 %)	6 (3.0 %)	0.078
Beans and nuts (Mean ± SD)	2.0 ± 1.5	2.1 ± 1.5	1.7 ± 1.	0.005
>3 days/week	66 (12.0 %)	45 (12.7 %)	21 (10.6 %)	0.497
Fruits and vegetables (Mean ± SD)	2.0 ± 1.5	1.8 ± 1.4	2.5 ± 1.7	<0.001
>3 days/week	84 (15.2 %)	36 (10.2 %)	48 (24.2 %)	<0.001
Animal products (Mean ± SD)	3.7 ± 2.1	3.3 ± 2.0	4.3 ± 2.0	<0.001
>3 days/week	252 (45.7 %)	129 (36.4 %)	123 (62.1 %)	<0.001
Alcoholic and Non-Alcoholic beverages (Mean ± SD)	2.4 ± 1.7	2.3 ± 1.6	2.5 ± 1.7	0.360
>3 days/week	114 (20.7 %)	63 (17.8 %)	51 (25.8 %)	0.029
Fats and oils (Mean ± SD)	1.0 ± 0.2	1.1 ± 0.3	1.0 ± 0.2	0.063
>3 days/week	0	0	0	0
Physical activity levels				
Vigorous	294 (53.3 %)	237 (66.9 %)	57 (28.8 %)	<0.001
Moderate	195 (35.3 %)	99 (28.0 %)	96 (48.5 %)	<0.001
Light	63 (11.4 %)	18 (5.1 %)	45 (22.7 %)	<0.001

Data presented as mean ± SD and/or n (%)

Over 70 % of the participants were less than 30 years of age (age range 18–36 years). Significantly; male students had lower mean BMI than their female counterparts. Five percent of the study participants were abdominally obese. Animal products, cereal and grains were the most frequently consumed food groups. Over 50 % of the participants had vigorous level of activity, from which 67 % of them were male students.

Presented in Table 2 is the Spearman’s nonparametric correlation coefficient of the study variables. Significantly, age correlated positively with body mass index (BMI), consumption of cereals and grains (CG); beans and nuts (BN); fruits and vegetables (FV) and total physical activity per week (TPAW) but negatively with meat and animal products (MAP). BMI had significant positive correlations with WC, CG, FV and non-alcoholic beverages (NAB) but negatively with fats and oils (FO). WC significantly correlated positively with roots and tubers (RT), MAP, FV and NAB.

**Table 2:** Spearman’s correlation coefficient of the study variables

Variable	BMI	WC	CG	RT	MAP	BN	FV	FO	ANAB	TPAW
Age	0.15^a^	0.05	0.16^a^	0.04	−0.10^b^	0.27^a^	0.13^a^	−0.01	0.08	0.13^a^
BMI		0.64^a^	0.09^b^	0.04	0.08	0.04	0.15^a^	−0.10^b^	0.15^a^	−0.06
WC			0.04	0.13^a^	0.13^a^	0.01	0.11^a^	−0.05	0.14^a^	−0.16^a^
CG				0.17^a^	0.26^a^	0.30^a^	0.10^b^	−0.10^a^	0.22^a^	0.17^a^
RT					0.19^a^	0.15^a^	0.14^a^	−0.16^a^	0.07	0.15^a^
MAP						0.02	0.11^a^	−0.14^a^	0.31^a^	−0.02
BN							0.08^b^	−0.05	0.13^a^	0.14^a^
FV								−0.08	0.38^a^	0.09^b^
FO									−0.03	0.03
ANAB										0.14^a^

^a^Correlation is significant at the 0.01 level (2-tailed). ^b^Correlation is significant at the 0.05 level (2-tailed). BMI = Body mass index, WC = Waist circumference, CG = Cereals and grains, RT = Roots and tubers, MAP = Milk and animal products, BN = Beans and nuts, FV = Fruits and Vegetables, FO = Fats and oils, ANAB = Alcoholic and Non-alcoholic beverages, and TPAW = Total physical activity per week

Factors predicting general overweight/obesity in a multivariable logistic regression model are presented in Table 3. Students who consumed fruits and vegetables > 3 times per week had a 2.6 risk of having overweight/obesity. Students who were vigorously active as well as those that had normal WC were less likely to become overweight/obese.

**Table 3:** Multivariable logistic regression of factors affecting general overweight/obesity

Variable	OR (95 % CI)	P value
Intercept		0.004
Age (≥30 years)	2.2 (0.6 - 7.9)	0.240
Male	0.4 (0.2 - 0.9)	0.024
Cereals and grains (>3 times per week)	1.4 (0.7 - 2.8)	0.401
Roots and tubers (>3 times per week)	0.5 (0.1 - 2.8)	0.458
Milk and Animal products (>3 times per week)	0.8 (0.4 - 1.6)	0.573
Beans and Nuts (>3 times per week)	0.7 (0.2 - 1.9)	0.430
Fruits and Vegetables (>3 times per week)	2.6 (1.2 - 5.4)	0.015
Fats and oils (>3 times per week)	NA	NA
Alcoholic and Non-Alcoholic beverages (>3 times per week)	0.7 (0.3 - 1.5)	0.330
Vigorous activity	0.3 (0.1 - 0.7)	0.004
Moderate activity	0.5 (0.2 - 1.2)	0.130
Abdominally obese	81.6 (18.8 – 354.8)	0.001

Cox and Snell = 0.20, Nagelkerke = 0.36

Shown in Table 4 are factors predicting abdominal obesity in a multivariable logistic regression model. Factors that predicted abdominal obesity were consuming roots and tubers; and alcoholic and non-alcoholic beverages > 3 times per week.

**Table 4:** Multivariable logistic regression identifying factors affecting abdominal obesity

Variable	OR (95 % CI)	P value
Intercept		<0.001
Age (≥30 years)	0.7 (0.01 - 50.11)	0.847
Male	0.0 (0.0 - 0.5)	0.017
Cereals and grains (>3 times per week)	0.9 (0.2 - 3.6)	0.835
Roots and tubers (>3 times per week)	8.0 (2.2 -10.1)	0.017
Milk and animal products (>3 times per week)	2.2 (0.5 - 9.2)	0.276
Beans and Nuts (>3 times per week)	1.0 (0.2 - 6.6)	0.992
Fruits and vegetables (>3 times per week)	0.6 (0.2 - 2.0)	0.421
Fats and Oils (>3 times per week)	NA	NA
Alcoholic and Non-Alcoholic beverages (>3 times per week)	8.2 (2.2 - 31.1)	0.002
Vigorous activity	4.8 (0.9 - 25.6)	0.067
Moderate activity	2.1 (0.5 - 9.2)	0.329
Overweight/obese	81.7 (17.6 – 378.2)	<0.001

Cox and Snell = 0.21, Nagelkerke = 0.64

## Discussion

We have investigated demographic, dietary habits and physical activity predictors of general and abdominal obesity in a sample population of university students. Foods from the cereals and grains as well as meat and animal product food groups were the most frequently consumed foods in this young adult population. Male students (compared with females), students with normal waist circumference (compared with those abdominally obese) and those engaging in physical activity (compared with those engaging in moderate and low physical activity levels) had lower odds of being overweight/obese (BMI). However, students had higher odds of being overweight/obese (BMI) if they consumed fruits and vegetables > 3 times per week (compared with those consuming less than 3 times per week). In addition, female students (compared to male students), those who consumed roots and tubers > 3 times per week (compared with those consuming < 3 times per week) and students consuming alcoholic and non-alcoholic beverages > 3 times a week (compared with those consuming non-alcoholic beverages < 3times per week) had higher odds of being abdominally obese.

We found that over 45 % of the students consumed foods from the milk and animal products > 3 times a week, making it the most frequently consumed food group in this young adult population. Due to urbanization and the nutrition transition, the diets of individuals from developing countries like Ghana could be said to be changing towards milk and animal products.

As expected and in agreement with the findings of Akarolo-Anthony (Akarolo-Anthony et al. 2013), a large proportion (37 %) of the students consumed foods from the cereals and grains making it the second most frequently consumed food group. Rice, a cereal has become a staple food in Ghana and is eaten in every home. It is the most commonly served food at most parties and celebrations.

The high consumption of cereals like rice and animal products like chicken has been strengthened by the springing up of fast food restaurants, whose major product offering is packaged chicken and rice meals (Akarolo-Anthony et al. 2013). Furthermore, foods like kenkey, banku and tuo-zaafi which are made from either corn or millet flour are the staple foods of most Ghanaians probably contributing to making foods from the cereals and grains the second most consumed food group.

In the present study, male students had lower odds of being generally overweight/obesity and abdominally obese compared to their female counterparts. This finding is in keeping with several studies conducted in developing countries (Mogre et al. 2014; Oghagbon et al. 2009; Olusanya and Omotayo 2011). Even though contrary findings have been reported from studies conducted in developed countries (Stewart-Knox et al. 2012), our findings could be attributed to the fact that a large proportion of male students engaged in vigorous physical activity than their female counterparts. In keeping with the literature that obesity is associated with physical activity (Mogre et al. 2014; Mogre et al. 2012; Bulló et al. 2011; Worthy et al. 2010) we found that students who engaged in vigorous physical activity had lower odds of being generally overweight/obese. The findings of this study in conjunction with previous studies highlight the importance of sex as well as physical activity in explaining relationships between general overweight/obesity, abdominal obesity and other interacting factors (Stewart-Knox et al. 2012).

A positive significant correlation was observed between the consumption of fruits and vegetables, BMI and WC. This relationship remained significant after adjusting for gender and age, as we found that students who consumed fruits and vegetables > 3 times a week had higher odds of having general overweight/obesity and abdominal obesity. The association between fruit and/or vegetable intake and adiposity in cross-sectional studies has been inconsistent (Tohill et al. 2004). While some studies have reported an inverse relationship (Bazzano et al. 2002; Lin and Morrison 2002; Serdula et al. 1996; Kahn et al. 1997; Trudeau et al. 1998) between fruit and/or vegetable intake and general overweight/obesity, others have either reported no association (Paterson et al. 2012; Lahti-Koski et al. 2002; Liu et al. 2000) or a positive relationship (Gillman et al. 1995). An important limitation of this study worth noting was the combination of fruits and vegetables in the data analysis. These food groups have varying energy contents. This makes it difficult to specifically determine the association of these food groups individually on adiposity. Future studies should ascertain specifics of consumption relative to total intake of fruits and vegetables (Tohill et al. 2004) to establish the relationship between fruits and vegetables and obesity in this sample.

From our correlation table the consumption of foods from the roots and tubers group increased with an increase in WC but not BMI. The consumption of foods (especially potatoes and French fries) have been shown to be associated with increased weight and diabetes (Halton et al. 2006).

Participants who consumed roots and tubers for more than 3 times a week were more likely to become abdominally obese. In addition, from our spearman correlation analysis the consumption of foods from the roots and tubers group increased with an increase in BMI but not WC. High consumption of root and tuber foods (especially potatoes and French fries) have been reported previously to be associated with increased weight and diabetes (Halton et al. 2006).

An important finding of this study was that the consumption of alcoholic and non-alcoholic beverages was associated with abdominal obesity. From our multivariable logistic analysis, students who consumed alcoholic and non-alcoholic beverages (which included soda, fruit drinks, lemonade, beer, etc.) > 3 times per week were several folds at risk of being abdominally obese. This finding is in keeping with several cross-sectional studies (Malik et al. 2006; Liebman et al. 2003; French et al. 1994) which have demonstrated a positive relationship between non-alcoholic beverage consumption and weight status, even though inconsistent findings have also been reported (Øverby et al. 2004).

It is worth noting the strengths and limitations of this study. This is a novel study in Ghana that assessed dietary habits, demographic and physical activity relative to BMI and WC in a sample of young adults. It also used a validated and comprehensive physical activity questionnaire, employing metabolic equivalents for calculating energy expenditure from physical activity. The findings of this study serve as a basis for future studies. It also adds to existing knowledge about the risk factors for general and abdominal overweight/obesity in a country undergoing nutrition transition. One of the limitations of this study is that it is cross-sectional and causality cannot be inferred from cross-sectional analyses because the data was collected at a single point in time and the direction of the association cannot be determined. Also we did not include socio-economic status of the participants in the analysis. Questions on socio-economic status were included into the questionnaire; however the responses were either missing or incomplete. More than 70 % of the participants declined from providing information on their daily and/or monthly income. Another limitation is that, all dietary data were obtained by means of a food frequency questionnaire. Food frequency questionnaires do not collect detailed information on the preparation or physical form of foods consumed which have been shown to affect the energy density of foods. This could have influenced the information relating to dietary habits and their association to other variables in the study. Portion sizes were not also measured which can have an influence on body weight. This study was conducted among a sample of university students who are highly educated. Participants may have had better recall of dietary intakes than the general population. Even though the sample population may be a true reflection of the urban population, it might not be representative of young rural Ghanaian adults who are not highly educated.

## Conclusion

Significant associations between demographic factors, dietary habits and physical activity levels were found. General overweight/obesity and abdominal obesity were less likely in male students and those engaging in vigorous physical activity. The consumption of fruits and vegetables >3 times a week was associated with general overweight/obesity. Furthermore, abdominal obesity was more likely in students who consumed roots and tubers > 3 times a week and alcoholic and non-alcoholic beverages > 3 times a week. Interventions should be designed by policy makers and health providers to promote active lifestyles.

## Abbreviations

WHO: World Health Organisation
GPAQ: Global Physical Activity Questionnaire
MET: Metabolic equivalents
UNICEF: United Nations International Childrens’ Education Fund
WHR: Waist to hip ratio
WC: Waist circumference
BMI: Body mass index
FFQ: Food frequency questionnaire
